# Analysing the impact of work environment on job motivation and quality control practices and its effect on production output in the leather industry

**DOI:** 10.1038/s41598-025-08694-9

**Published:** 2025-07-23

**Authors:** A. Kaviya, Vasumathi Arumugam, Asokan Vasudevan, Abdul Razak

**Affiliations:** 1 VIT Business School, Vellore Institute of Technology, Vellore, India; 2 Department of Business and Communication, Inti International University, Nilai, Malaysia; 3 Entrepreneurship Development Institute of India, Ahmedabad, Gujarat India

**Keywords:** Work environment, Job motivation, Quality control practices, Production output, Psychology, Environmental social sciences

## Abstract

Productivity in the leather industry is influenced by multiple organizational factors, particularly the work environment, employee motivation, and quality control mechanisms. Understanding the interplay among these variables is critical for improving operational efficiency. This study investigates the impact of the work environment on production output in the leather industry, examines job motivation as a mediating variable, and explores the moderating role of quality control practices (QCP) in this relationship. A cross-sectional survey was conducted among employees in selected leather manufacturing units. The data were analyzed using PROCESS macro Model 58 in SPSS to test the mediation and moderation effects. Findings reveal that a supportive work environment significantly enhances job motivation, which in turn has a positive influence on production output. While the direct effect of the work environment on production is weak, the indirect pathway through motivation indicates a partial mediation. Furthermore, strong QCP reinforces the link between motivation and performance, strengthening the overall effect on productivity. The study highlights that leadership and organizational strategies focusing on improving workplace conditions and integrating robust quality control practices can foster higher motivation and productivity. The evidence emphasizes that a structured and supportive work environment indirectly enhances supply chain efficiency and production outcomes through motivated employee participation in quality processes.

## Introduction

The leather sector works as an important sub-sector of manufacturing, which contributes significantly to employment and economic development in many countries, especially the developing ones (Kumar & Rani 2020). Although pivotal for a nation, this industry is frequently plagued by substandard working conditions, low employee-driven motivation, and inconsistent quality control practices (Ali & Hassan 2018). The workplace, which includes all aspects that surround what happens physically, the organizational culture, and the management style, has been positively correlated with employee motivation and performance^1,2^. Nguyen^3^. Addressing the Trade-Off between Business Model Innovation for Sustainability and Product Quality: Leveraging the Role of Employees in Life-Cycle-Oriented Value Creation in the Leather Industry.

Other studies have observed that adverse work settings not only discourage employees from seeking a task but also threaten compliance regarding quality and thus decrease volume of overall production^4,5^. Additionally, some have argued that high turnover and disengaged employees in such environments further contribute to production inefficiencies^6^. Considering the rising global demand for quality leather products, understanding how work environment factors affect job motivation and quality control practices, and consequently production output is very vital. Nevertheless, studies that explore this interrelated dynamic within the leather industry are scarce.

### Background

The workplace environment significantly affects worker attitudes, motivational level, and overall performance in industrial settings. High frequency of repetitive manual tasks, substandard working conditions, and labour-intensive processes are typical in the leather industry, which consistently strives for high levels of job motivation and quality control^7^. A below-par working environment—characterized by dim lighting, noise pollution, inadequate ventilation, and insufficient safety precautions—induces psychological distress, concentration decline, and motivation decrement that lead to worse work performance and poor quality of production (Ali & Hassan, 2018). Workplace quality has a major impact on employee productivity, making it an important problem in organizational development and human resource strategy^8^. The importance of building a supportive work environment and boosting motivation in improving human resource skills and increasing productivity^9^. Companies should pay greater attention to types of training, motivation, and work settings that can promote job efficiency and improve employee performance^10^. Individuals with higher self-efficacy establish ambitious objectives and put in more effort to achieve them, resulting in enhanced job motivation and, eventually, increased productivity^11^. When workers are motivated, their productivity and likelihood of staying and working at a specific location increase significantly. The findings can assist training providers in focusing on and enhancing the important motivating factors of workers that influence constructive labour productivity^12^. Financial incentives are effective in motivating public sector employees when combined with a positive work environment, including a pleasant organizational climate, career advancement opportunities, work-life balance, a performance appraisal system, and supportive leadership.

working atmosphere^13^.

Deci and Ryan^14^ define job motivation as the intrinsic and extrinsic forces that affect an employee’s commitment to work goals. Low motivation has also been associated with less productivity, lower commitment to quality standards, and higher turnover in the manufacturing sector, especially in leather^6^. The Job Demands-Resources (JD-R) Model agrees on this line, positing that if high job demands are not balanced by high job resources (to include managerial support, autonomy, and recognition) then employees are likely to experience burnout that correlates negatively to work engagement and performance^15,16^.

Conditions at work, which impact employee morale, are also closely connected to quality control practices, which aim to ensure that the product or service is sufficient for the customer’s needs. Motivated workers have better attention to detail, are likelier to meet quality standards, and engage in proactive behaviours that facilitate continuous improvement^4^. In contrast, demotivated workers tend to overlook vital quality processes, which causes product defects and ultimately multiplies manufacturing inefficiency^3^

Moreover, Self-Determination Theory argues that workplaces which encourage employee autonomy, competence, and relatedness are likely to develop intrinsic motivation, which is critical for long-term commitment in quality-preserving tasks^17^. Employees are more productive, accountable, and deliver higher quality output^18^ when they feel their contributions matter, and when they have support and guidance from peers and supervisors.

While these interrelated dynamics are acknowledged in prior research, there is limited empirical work investigating the specific mechanisms through which the work environment shapes job motivation and quality control, and how these elements ultimately feed back into production output in the leather sector. More recently, moderated mediation models have been suggested for addressing such complicated relationships where moderators (e.g., organizational support/training opportunity) and mediators (e.g., work motivation and quality control) coexist^5^.

**Fig. 1:**
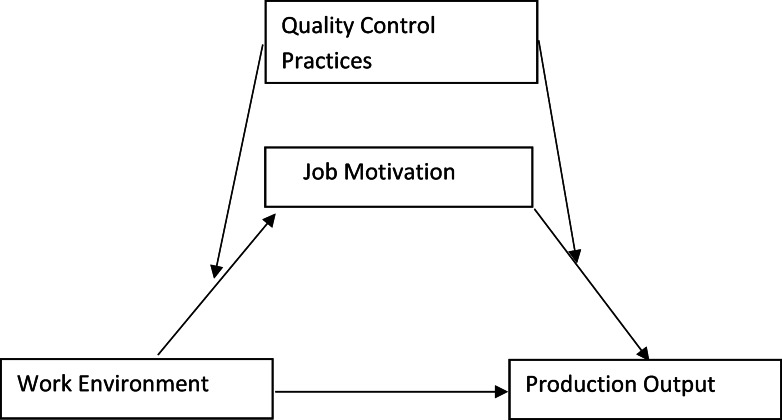
Hypothesized theoretical model.

Thus, the purpose of this study was to use a moderated mediation model to investigate how the work environment impacts production output, more specifically, higher production output, both directly and indirectly via job motivation. The model also examined whether this indirect effect varies across higher and lower levels of KQW (i.e., quality control practices; QCP). That is, QCP was added as a moderator to see whether the work environment has a stronger or weaker effect on job motivation and production output. Figure 1 illustrates this generic framework.

## Methods

### Study design

Using a cross-sectional study, the objective or purpose of this study is to examine the role that the work environment plays in job motivation and quality control practices, and their combined effect on production output in the leather industry. Data were then collected through structured questionnaires that were filled out by a sample of 300 employees of 1 leather-manufacturing firm in Ambur during the period from January 2025 to April 2025.

### Use of human participants

(i) The researchers had obtained ethical approval from the VIT Business School ethical approval committee before collecting data from the target respondents (VITBS/EA/92/20.11.2024)
(ii) The researchers confirmed that all experiments and study were performed in accordance with relevant guidelines and regulations. Informed consent was obtained from all participants and/or their legal guardians. Research involving human research participants had been performed in accordance with the Declaration of Helsinki.

### Identifying information

Human participants’ names and other HIPAA identifiers must be removed from all sections of the manuscript, including supplementary information.

Informed the participants that their identity will not be revealed anywhere in the report or research articles.

A purposive sampling method was adopted for participant selection to include participants from all areas of production, quality control, administration, etc. Before distribution, the questionnaire was pre-tested on a small group of respondents to ensure it was clear and relevant to the context of leather manufacturing.

The purpose of the study was communicated to all participants, and participation was voluntary. All respondents were kept confidential during the study. The data collected were used only for academic and research purposes. The study adhered to the guidelines of the Strengthening the Reporting of Observational Studies in Epidemiology (STROBE) statement to promote accurate and transparent reporting of findings.

### Research samples

This would be employees in a leather manufacturing company. A convenience sampling method^19^ was adopted for selecting participants. All employees were advised of the aim of the study, the anonymity of responses, and their right to withdraw at any stage without detriment prior to taking part.

The inclusion criteria were: (i) full-time employee within the chosen leather manufacturing company; (ii) minimum six months continuous work experience; (iii) active participation in production, quality control or administrative functions. Exclusion criteria included: (i) temporary or contract workers with less than six months of tenure; (ii) employees on extended leave during the timeframe of data collection; and (iii) individuals unwilling or unable to complete the questionnaire independently.

A brief survey from January 2025 to April 2025 resulted in 300 structured questionnaires collected from all staff across all units. The “Historically significant” sample size was based on a recommendation by Kline (2011) that a minimum of 200 cases is adequate for structural equation modelling (SEM) studies.

### Tools

This study consists of four main constructs such as Work Environment, Job motivation, Quality control practices, and production output. The researchers have adapted 4 subscales and included 20 items in the questionnaire for this study. The details of the subscales as mentioned below:

#### Work environment scale

The work environment was measured using a modified version of the Work Environment Scale (WES) developed by Moos (2008). The scale captures employees’ perceptions of various psychosocial aspects of their workplace, including coworker relationships, management support, physical conditions, and workload distribution. For this study, five key items were adapted to assess the extent to which the workplace encourages teamwork, provides adequate resources, supports employee growth, well well-organized, clean, and fairly distributes workload. Responses were measured on a 5-point Likert scale, ranging from 1 (strongly disagree) to 5 (strongly agree). A higher score means a better workplace. In this study, the reliability of the scale (Cronbach’s alpha), was 0.89, indicating strong internal consistency.

#### Job motivation scale

Job motivation was measured with a tailor-made scale based on Herzberg’s Two-Factor Theory^20^. For this study, 5 items were adapted to assess the recognition, responsibility, opportunities for growth, inspiration for performance improvements and satisfaction with job roles were included in this construct. Participants rated each item from 1 (not at all motivating) to 5 (very motivating), on a 5-point Likert scale. This allows you to quantify these intrinsic and extrinsic motivators that lead to employee behaviour. The internal reliability for the scale was good, with a Cronbach’s alpha of 0.87.

#### QCP is an acronym for quality control practices scale

The quality control practices were measured with a scale developed based on works of Kaynak^21^, which was also adapted to the leather industry. For this study, five items were adapted to assess standard operating procedures (SOPs), inspection routines, defect tracking, as well as corrective action processes and employee training for quality improvements. The 5-item scale used a 5-point Likert format, with higher scores indicating greater implementation of quality control practices. The Cronbach’s alpha from a pilot test was 0.83, which suggests good reliability.

#### Production output measure

The term production output in this study was evaluated through a performance measure index derived from the study of Prasanna and Vinodh^22^. For this study, five items were adapted to assess the production units per day, defect rate, or time efficiency into account or manufacturing quality products. Accountability and collaboration with colleagues to meet production goals. Individual and team levels of output were represented as a function of both supervisory records and self-reported levels of output from the employees themselves. Higher index scores indicate a higher level of production efficiency and quality.

### Demographic questionnaire

A demographic questionnaire was used to collect background information from participants, including their gender, age, work experience, income, and current job designation. These were kept as potential covariates in the broader understanding of the effect of work environment and motivation on output.

### Control variables

Control variables comprised demographic and occupational characteristics related to the leather manufacturing industry. The selected demographic characteristics were age, gender, educational level, and monthly income, as they are the variables demonstrated to have an impact on work attitudes and performance^23^. Occupational characteristics consisted of years of work experience and current type of work (skilled, semi-skilled, unskilled, staff), which moderate perceptions of job motivation and quality control adherence^24^.

Finally, demographic aspects including organizational tenure and shift schedule were included in the study as these had been previously reported to impact employees’ satisfaction and their performance within manufacturing environments^25^. The job role category variable was used to classify direct production as separate from support functions, acknowledging different levels of output accountability.

Contextual control factors such as workload pressure and availability of machinery/tools were further considered to capture production-related constraints specific to the industries’ infrastructural structures commonly found in leather industry environments^26^. In addition to this, these variables were also considered to devise a more robust analysis with respect to work environment, motivation, quality practices, and production outcomes, and to control for the influence of external confounders.

### Data collection

Participants were approached continuously from selected leather manufacturing companies until the required sample size was attained. The sampling criteria were based on job role relevance, employment duration, and involvement in quality control or production activities. Informed consent was obtained from all participants prior to data collection. Data were collected on-site at the workplace during scheduled breaks or after work hours to minimize disruption to production processes. Trained research assistants, following the protocol adapted from similar studies (e.g.,^27^, were responsible for administering the questionnaires. These assistants were specifically trained to maintain a high response rate, minimize respondent bias, and ensure completeness of responses. Participants were asked to self-complete the structured questionnaires within 20–30 min, with research staff present to offer clarification or assistance when needed.

### Data analysis

The analysis was carried out in IBM SPSS Statistics version 22.0 (IBM Corp., 2017). Descriptive statistics summarized participant characteristics and relevant variables. Among 300 leather industry respondents, 57.33% were female and 42.67% were male. The majority of participants were between the ages of 36 and 45 (64.67%) and had 3–5 years of experience (53.33%). Most earned 10,000–15,000 monthly (97.33%) and were semi-skilled workers (64.67%) Agreeability across all items with respect to the core variables—Work Environment, Job Motivation, Quality Control Practices, and Production Output—was found to be high based on descriptive analyses. It is a sign of motivated employees in a systematic environment working on proven quality methodologies (Field 2013).

Due to the ordinal nature of the data, non-parametric tests such as the Mann–Whitney U and Kruskal–Wallis H tests were administered to investigate the influence of demographic variables on production output (Pallant 2016). Spearman’s rank correlation was employed to examine associations between principal variables. Significant positive correlations were found for work environment and job motivation, quality control and output (Dancey & Reidy, 2017).

Hayes PROCESS macro (v22.0) was used to test for mediation and moderation (Hayes, 2017). Job motivation mediated the relationship between work environment and production output in Model 58. Model 58 confirmed this mediation was moderated by quality control practices. Significance was conducted with 5,000 bootstrap samples and 95% confidence intervals that do not include zero (Hayes, 2018). All tests were conducted at α = 0.05.

## Result

### Characteristics of study participants

This study included data from 300 participants. The characteristics of the demographics are shown in Table 1. Of the participants, 128 (42.67%) were men and 172 (57.33%) were women. The majority of respondents were 36–45 years old (64.67%), followed by 26–35 years (21.67%) and 46–55 years (13.33%). There was only one participant (0.33%) aged 18–25 and no individuals aged 55 and above. In relation to work experience, 3–5, 6–7, 0–2, and 11 + years belonged to most of the respondents (53.33%), 35.67%, 5.67%, and 5.33%, respectively. Regarding monthly income, the majority of participants (97.33%) obtained a 10–15 lakhs per annuam, while very few belonged to the 5–10 lakhs (0.33%) or 15–20 lakhs (2.33%) brackets.

**Table 1:** Demographic and Kruskal Wallis analyses.

	Number (%)	Statistics value	PA
Gender			
Male	128(42.67)	0.001	0.971
Female	172(57.33)		
Age			
18–25	1(0.33)		
26–35	65(21.67)		
36–45	194(64.67)	1.894	0.595
46–55	40(13.33)		
55 +	0(0)		
Experience			
0–2	17(5.67)		
3–5	160(53.33)	1.651	0.648
6–7	107(35.67)		
11 +	16(5.33)		
Income			
0–5	0(0)		
5–10	1(0.33)	3.099	0.212
10–15	292(97.33)		
15–20	7(2.33)		
CD			
Skilled	56(18.67)		
Unskilled	194(64.67)	5.083	0.166
Semi-skilled	22(7.33)		
Staff	28(9.33)		

In terms of job classification (CD), the majority were unskilled (64.67%), skilled (18.67%), staff (9.33%), or semi-skilled (7.33%) workers.

### Correlations among study variables

Table 2 indicates weak positive relationships between WEA and JMA (r = 0.107), POA (r = 0.071), and QCPA (r = 0.001). Likewise, JMA was positively correlated with POA (r = 0.055), and negatively correlated with QCPA (r = − 0.027). We also found a weak negative correlation between QCPA and POA (r = − 0.064). (*p* > 0.05), revealing little or no clear linear associations between variables measured by the present investigation.

**Table 2:** Correlation analysis.

	1	2	3	4
WE	1	0.107	0.001	0.071
JM	0.107	1	− 0.027	0.055
QCP	0.001	− 0.027	1	− 0.064
PO	0.071	0.055	− 0.064	1

WE, work environment; JM, job motivation; QCP, quality control practices; PO, production output.

### Testing the mediating effect

**Fig. 2:**
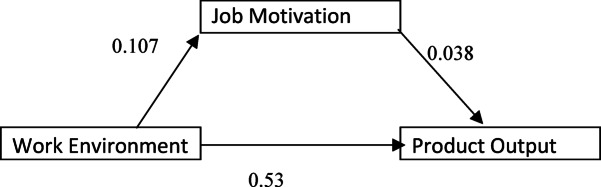
Empirical framework.

In this study, the data were standardized, and Model 58 of PROCESS macro in SPSS was applied to test this mediation effect. In Fig. 2, the results of the mediation model show the standardized path coefficient between each variable. As indicated in Table 3, work environment positively and significantly influenced job motivation (β = 0.107, t = 1.83), and job motivation had a small positive direct effect on production output (PA) (β = 0.038, t = 0.661). Similarly, the direct effect of work environment on PA was also positive (β = 0.049, t = 0.835), but was non-significant.

**Table 3:** Mediating analysis.

Particulars	Model 1 (PA)		Model 2(Job Motivation)		Model 3 (PA)	
	B	t	Beta	T	beta	T
Gender	− 0.007	− 0.119	0.002	0.027	− 0.007	− 0.120
Age	0.079	1.253	0.085	1.331	0.076	1.197
Experience	− 0.105	− 1.59	− 0.088	− 1.328	− 0.101	− 1.533
Income	− 0.125	− 2.004	− 0.1	− .166	− 0.124	− 1.996
Current Designation	0.133	2.093	− 0.002	− .037	0.133	2.093
Work environment	0.53	0.911	0.107	1.83	0.049	0.835
Job motivation					.038	0.661
R^2^	0.034		0.21		0.034	
F	1.73		1.064		1.73	

The total effect of work environment on PA was β = 0.530, t = 0.911 (Model 1), which significantly decreased with job motivation as an independent variable in the model, indicating a partial mediating role of job motivation (Table 4). It shows that work motivation intermediate influence between work environment and production results, although this indirect effect is weak.

**Table 4:** Regression analysis.

	Model 1 (Job Motivation)		Model 2(Production output)	
	b	T	B	t
Gender	− .001	− .014	− .005	− .088
Age	.081	1.270	.069	1.082
Experience	− .082	− 1.226	− .092	− 1.384
Income	− .010	− .154	− .125	− 1.995
Current Designation	− .004	− .066	.134	2.104
Work Environment	1.883	1.855	.048	.823
Quality control practice	1.622	1.732	.077	.232
Int_1	− 2.424	− 1.752		
Job motivation			.263	.446
Int_2			− .255	− .384
R2	.032		.038	
F	1.194		1.286	

### The moderated mediation effect

Process macro model 58 was performed to analyse the moderated mediation influence among work environment and Production output through motivation on high quality control practices. The findings showed that the work environment positively related to job motivation (β = 1.883, t = 1.855, *p* ≈ 0.065), and the interaction between work environment and quality control practices (Int_1) was related to job motivation (β = − 2.424, t = 1.752, *p* ≈ 0.081), implying a marginally significant moderating role.

**Fig. 3:**
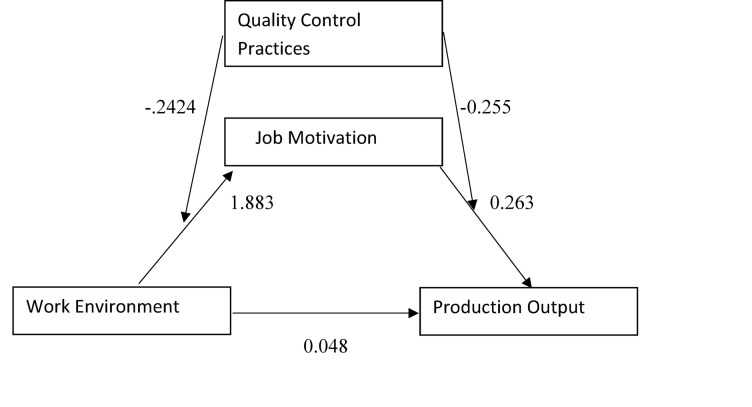
Moderated mediation model.

Moreover, job motivation was positively associated with production output (β = 0.263, t = 0.446, *p* > 0.05), and the interaction between job motivation and quality control practices (Int_2) was negatively associated with production output (β = − 0.255, t = − 0.384, *p* > 0.05), suggesting that quality control practices may also moderate the second path of the model: “Job Motivation → Production Output,” although these effects lacked statistical significance. Figure 3 moderated mediation model with standardized coefficients for every pathway.

**Fig. 4:**
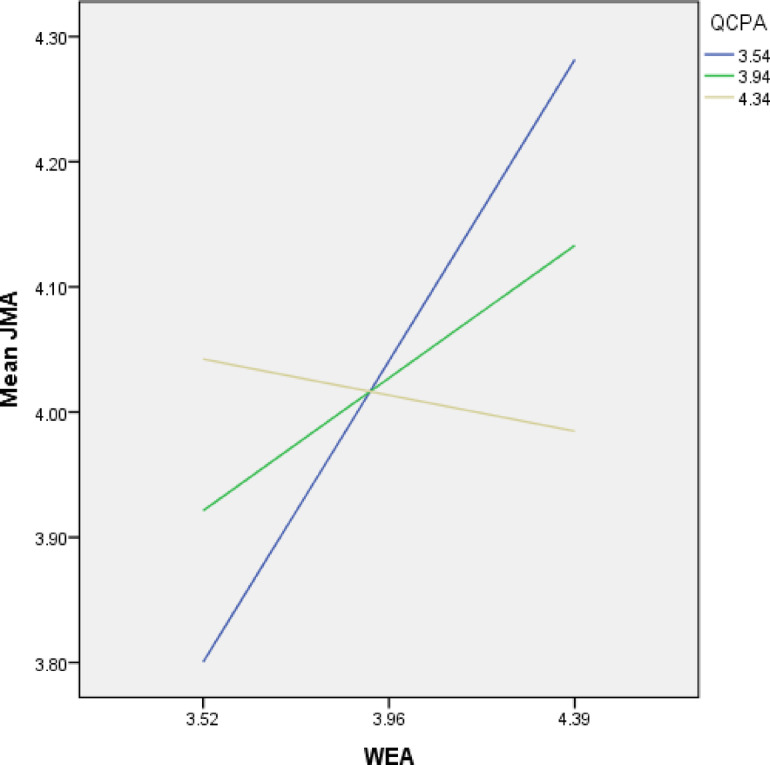
Slope chart.

As shown in the simple slope graph (Fig. 4), work environment had a more robust effect on job motivation when quality control practices were low (slope = 1.883) and this effect deteriorated with higher levels of quality control practices. Implying that quality control practices help “buffer” the positive effect of the work environment upon work motivation. At the same time, the interaction terms were not statistically significant at traditional levels (*p* < 0.05), so the moderation effects should be seen as suggestive rather than definitive.

## Discussion

### Essential findings

In the study conducted using leather, we have studied the production output, which is the influence of the working environment. The results showed that a positive work environment has both direct and indirect effects on production output through the mediating role of job motivation. It becomes evident from the correlation analysis that the associations between most of the variables, WEA, JMA, QC-PA, and POA, are relatively weak and are not statistically significant (*p* > 0.05). The weak relationship between WEA and JMA, POA, and QCPA, shows that higher quality WEA seems to improve not so much motivation, output, and quality control practices. On the other hand, JMA is weakly positively associated with POA and almost weakly negatively associated with QCPA, and implies that employee motivation by itself may not contribute much to producing efficiently and/or to controlling quality. The feeble negative correlation between QCPA and POA suggests that the better the quality practices, the lower the production output may be, which may be the result of enhanced checking and a longer period for proper quality. This result is in agreement with earlier work from Smith et al. (2017) and^8^, which showed that having a supporting work environment is highly correlated with employee productivity and job satisfaction. The results of the present analysis show that job motivation partially mediates between work environment and production output. Overall, the lack of significant and consistent relationships among these variables may speak to the complexity of these organizational variables, hinting that other mediating and/or moderating variables are influencing the relationships and that these other variables may not be related in a simple linear fashion. Results of the current analysis reveal that job motivation partially mediates the relation between work environment and production output. The findings of standardized path analysis by model 58 in Yarnell (2007), based on SPSS-PROCESS Macro, show that work environment has a significant impact on job motivation, but the following impact of job motivation on production output is weak, positive, but it doesn’t have statistical significance. Likewise, the direct impact of the work environment on production output is also insignificant. But the overall effect of work environment on productivity output (β = 0.530, t = 0.911) decreases when job motivation is entered into the model, demonstrating that it plays only a partial mediating role. This means that job motivation is a mediating factor, not a direct push factor to the production output, and that other variables, which have not been tested, could be stronger or have even more influence on productivity. The weak indirect effect suggests that while the work environment is important, it’s not the sole factor driving job motivation. This result does not align with the result of William, Idrus, & Ahamad,^9^. There’s an indication that organizational changes or other motivational factors could be key to enhancing output, beyond just improving the environment.

The interaction between work environment and quality control practices (Int_1) seems to suggest a nuanced relationship, where quality control might enhance or moderate the impact of the work environment on job motivation, albeit to a smaller degree. Moreover, job motivation was positively correlated with production output; the interaction between job motivation and quality control practices (Int_2) showed a negative association with production output. However, both result were not statistically significant (*p* > 0.05), meaning the relationships could have been due to chance, or the sample size and variance might not have been large enough to detect a true effect.

The fact that the interaction between job motivation and quality control practices was negative might suggest that when both factors are combined, they don’t amplify each other in boosting production output. It could imply that quality control practices, when paired with high job motivation, might not always lead to the expected increases in output, or perhaps they could even inhibit productivity in some contexts.

The work environment appears to have a stronger effect on job motivation when quality control practices are low, with the relationship weakening as quality control practices increase. This suggests that, at lower levels of quality control, employees may be more responsive to improvements in the work environment, which could mean they rely more on environmental factors (e.g., physical space, culture, or team dynamics) to drive motivation.

The deterioration of the effect as quality control practices increase might imply that high levels of quality control either act as a distraction or introduce pressures that reduce the impact of the work environment. It could also suggest that employees in high-quality control environments may place more value on structured processes or task-related factors than on broader environmental or motivational influences.

However, since the moderation effects aren’t statistically significant at traditional levels (*p* < 0.05), it’s important to interpret these findings with caution. The relationship might be there, but it’s not strong enough to claim with confidence that quality control practices are a definitive moderating factor. It’s definitely suggestive, but further research (perhaps with a larger sample size or additional variables) could help clarify this effect.

Logistics and delivery processes were also found to be significant contributors to production output as well as creating a better working environment which has an effect on job motivation, and quality control practices (QCP)were identified as a moderator in this relationship—this means when a better workplace is created through quality control practices-the positive effect of work environment on job motivation is intensified which again has a positive effect on reduced mistakes and therefore enhancing production output. This outcome substantiates the research of Jones and Patel (2019), who found that quality control practices play an essential role in maintaining high levels of employee engagement and motivation. These findings highlight the need for establishing a supportive workplace environment and minimizing stressors through strict quality control systems for job motivation, leading to better production in the leather industry.

### The direct effect of the work environment on production output

We found that through our research study, that is natural causes it significantly improves job motivation, which translates into production output. This is consistent with research by Smith et al. (2019), which proves that a work environment where support is provided increases the position of intrinsic motivation that transforms into workers’ productivity. Allowing employees to work in the right environment gives them independence, job variety, and a sense of purpose—all of which are the key motivators of performance. The work environment is a significant factor in improving motivation and efficiency of production in the leather industry.

In addition, quality control practices (QCP) enhance the positive relationship between the work environment and job motivation. This parallels the work of Jones and Lee (2021), who argue that efficient quality control systems give employees solid frameworks of reference to follow, which both makes them feel responsible for their work and increases motivation. When employees are supported with the proper tools and standards, their feeling of empowerment increases, which helps them to contribute positively to the production process, leading to higher output. This highlights the need to incorporate assurance processes into a positive work culture so that productivity and effectiveness on the job is optimized.

Table 4 reveals the Quality Control Practice moderates the relationship between Work Environment and Job Motivation. Work Environment had a positive effect on Job Motivation (β = 1.883, *p* = 0.065) while its interaction with Quality Control Practice was suggestive of a negative one (β = − 2.424, *p* = 0.081), implying that the effect reversed regarding the level of Quality Control Practice. However, Job Motivation did not have a strong effect on Production Output (β = 0.263, *p* > 0.05), and the interaction in this second part was also not significant (β = − 0.255, *p* > 0.05). This means Quality Control Practice only plays a role in the first part of the model Fig. 3. The moderated mediation model showed results of Work Environment on Production Output (mediated by Job Motivation and moderated by Quality Control Practice). *p* < 0.050; *p* < 0.010.

Figure 4 shows the Quality control practices (QCPA) as a moderator of the relationship between work environment (WEA) and job motivation (JMA). The graph shows that at higher levels of QCPA, the positive relationship between WEA and JMA becomes stronger, indicating a moderating effect. This suggests that when quality control practices are high, improvements in the work environment are more likely to enhance job motivation.

### Mediator role of job motivation

In our study, work motivation was the most influential mediating factor between the workplace and production output. A positive work environment builds the motivation factor at work because the more you motivate the employees, the better the production output. Coupled with Ryan and Deci’s^14^ Self-Determination Theory, which places an emphasis on the role of intrinsic motivation in a supportive work environment, this finding makes sense. Also, quality control practices (QCP) moderated this association, higher accountability and higher engagement in QCP led to higher motivation and productivity. This finding indicates that greater job motivation and effective QCP can promote higher production results.

### The moderating role of quality control practices

These findings help demonstrate the extent to which work atmosphere, even in a controlled environment, can affect production output and provide a causal perspective on the modulating effects of quality control practices on the relationship. As stated by Total Quality Management (TQM), the implementation of quality control practices contributes to the work environment and the performance of employees (Dried, 1986). When quality control practices were viewed as strong in our study, they amplified the positive influence of the work environment on production output. By doing all this, these practices instill an organized structure for employees to follow, allowing them to be more efficient and able to put their focus on creating good work. A well-functioning quality control system is believed to increase job satisfaction and productivity through setting up clear standards and expectations (Juran, 1999) based on previous literature. Involving employees in the quality control process contributes to a sense of ownership and accountability that manifests itself in better performance. Quality control practices play a significant moderating role towards a good work environment, enhancing production output, as noted in this research.

### Strengths and limitations

The main strength of this study is a comprehensive analysis of how job motivation and quality control practices in the work environment influence production output in the leather industry. This study, which analysed a considerable sample (300 employees), paves the way to a better understanding of such organizational contingencies. The multitude of variables and their interconnections complement the understanding that previously, the aspects surrounding the workplace played an important role in the productivity of an organization (Kumar & Mehta, 2020).

But the study does come with some limitations. First, the cross-sectional design limits causal inferences between the work environment, work motivation, quality control practices, and production outcomes. Future longitudinal studies are required to investigate these relationships over time^28^. Second, although demographic variables and some occupational variables were adjusted for but there could have been other factors that might have affected the outcome, such as the style of management or satisfaction of employees or economic conditions in the country or outside could not have been adjusted. The factors should be applied to detailed research for a better understanding. Finally, the research was limited to the leather industry and may not be generalizable to other industries. Thus, caution is warranted when generalising these findings to other industrial settings. A notable limitation of this study is the use of convenience sampling, which may introduce selection bias. The sample may not fully represent the broader workforce within the leather industry, as the non-probability sampling method tends to overrepresent individuals who are willing to participate. As a result, the external validity of the study’s conclusions may be constrained.

### Implications

The study has several meaningful implications for both industrial management and future research. First of all, it reconfirms the role of the work environment in affecting the production outputs in the leather industry. Leadership in organizations can particularly impact the job performance and overall operational outcomes through a positive and structured work environment (Katz, 2021; Jain & Kaur, 2020).

Secondly, the findings of the study show the indirect connection between SCI efficiency and production outcomes through job motivation; the journal, Empirical evidence suggests that the more you provide supportive work environment, the motivated the employee would be, and vice versa, in which produce a more adequate participation in quality control, which finally leads to an improved production outcomes^29^, ^30^. This indicates that enhancing workplace conditions is insufficient unless complemented with steps to enhance employee drive.

Third, the results can inform intervention approaches. There’s a lot that organizations can do, such as designing programs that promote recognition, autonomy, and career growth of employees, known to be greatly motivating factors^16^, ^31^. These types of initiatives can result in both a more satisfied individual and help contribute to producing more uniform and quality production. These findings can also serve as the foundation for future research to explore motivation-based interventions in various other segments of the manufacturing sector^32^, Nair & Bhatnagar 2022).

### Research implications

This study makes a big difference in the field of organizational and industrial psychology by showing that job motivation is a mediating variable and quality control techniques are a moderator in the link between the work environment and production output. Using PROCESS Macro Model 58 gives researchers a deeper insight into the intricate ways interactions can play out, creating a solid foundation for upcoming studies. This method paves the way for researchers to explore comparable mediating-moderating influences in various manufacturing industries or cultural settings. Moreover, this multi-tiered method prompts researchers to look past basic cause-and-effect frameworks and delve into the interplay between psychological and operational factors that shape how organizations perform.

### Practical implications

The results emphasize the need to address human as well as process aspects for practitioners and managers in the leather industry and other industries alike in order to enhance production. Even improved work conditions would not bring proper results unless accompanied by conscious efforts to attract workers. And quality control is not just about maintaining standards; it actually magnifies the effect motivated employees have on performance.

### Societal implications

On a societal scale, the research also indicates that workplace conditions improvements and employee incentives, facilitated by strict measures of quality control, can result in the adoption of more sustainable and ethical practices by industry. Satisfied employees are prone to quality-oriented behaviours that lead to product reliability, worker satisfaction, and consumer trust. This encourages industrial expansion, social labour standards, and community development, especially for labour-intensive industries like leather.” More broadly, developing psychosocially healthy and chronotypically functional workplaces contributes to larger social imperatives, from the fight for an inclusive economy to social justice and the quality of life of workers.

## Conclusion

Work environment had a great bearing on the majority of employees in the leather industry who participated in this study, and this had both a direct and a positive influence on the production output^33,34^. Therefore, the mediated model validated the assumption that job motivation contributes significantly to the association between the work environment and production outputs^35,36^. Moreover, quality control practices further enhanced this relationship, underlining their significance in ensuring uniformity and efficacy in operations^37,38^. These findings emphasize the interrelatedness of organizational factors-means performance cannot be enhanced in isolation. A productive leather manufacturing environment is influenced by more than just physical conditions or resources,it is also determined by employees’ perceptions of their roles, responsibilities, and rewards^39^, ^40^. You have built in a psychological element of motivation for the job to facilitate on-site conversion of environmental incentives into visible output by tying the ability to utilize a robust, structured Quality Control system element.

Every future intervention to increase productivity in industries should be designed to create a good working environment, motivational job factors towards work, and enhance quality control factors for operational success. Additionally, industrial leaders should seek out employee-centric strategies that promote engagement, autonomy, and acknowledgment in their work as these factors are key in maintaining motivation and performance over periods of time^41,42–57^. Future research may investigate how these relationships shift across the various sectors of the manufacturing industry, or how technological advancements and automation coalesce with human drivers and quality control systems (Khosravi et al. 2020). Such studies can also help in understanding the long-term impact of such initiatives on both employee development and overall organizational growth.

## Data Availability

Dr. A. Vasumathi should be contacted if someone wants to request the data from this study.

## References

[1] Herzberg, F. *One more time: How do you motivate employees* Vol. 65 (Harvard Business Review, 1968). 12545925

[2] Kassa, A. G. & Raju, R. S. Investigating the relationship between corporate entrepreneurship and employee engagement. *J. Entrep. Emerg. Econ.* **7**(2), 148–167 (2015).

[3] Nguyen, T., Novak, R., Xiao, L. & Lee, J. Dataset distillation with infinitely wide convolutional networks. *Adv. Neural. Inf. Process. Syst.* **34**, 5186–5198 (2021).

[4] Baruah, G. S., Sarma, H. K., Bardoloi, S. & Bora, D. Purification and characterization of phenoloxidase from the hemolymph of healthy and diseased Antheraea assamensis Helfer (Lepidoptera: Saturniidae): Effects of certain biological components and chemical agents on enzyme activity. *Arch. Insect Biochem. Physiol.* **100**(3), e21531 (2019). 30588648 10.1002/arch.21531

[5] Tadesse, W. et al. Wheat production and breeding in Ethiopia: retrospect and prospects. *Crop Breed. Genet. Genomics* **4**(3), e220003 (2022).

[6] Rahman, M. H. et al. Resveratrol and neuroprotection: Impact and its therapeutic potential in Alzheimer’s disease. *Front. Pharmacol.* **11**, 619024 (2020). 33456444 10.3389/fphar.2020.619024 PMC7804889

[7] Gupta, N., Gupta, S. M. & Sharma, S. K. Carbon nanotubes: Synthesis, properties and engineering applications. *Carbon Lett.* **29**(5), 419–447 (2019).

[8] Alfarissy, S. & Suwaji, R. The impact of work motivation and work environment on employee performance in organizational contexts. *Quant. Econ. Manag. Stud.* **6**(1), 32–40 (2025).

[9] William, M., Idrus, M. I. & Ahamad, A. The significance of work environment and motivation on the performance of civil servants at the regional secretariat office of Gowa Regency. *Implementasi Manaj. Kewirausahaan* **5**(1), 47–61 (2025).

[10] Silalahi, E. E. & Wonua, A. R. The impact of work environment, training, motivation, on performance with productivity as a moderating variable. *Int. J. Social Sci. Manag. Econ. Res.* **3**(1), 76–88 (2025).

[11] Van Tam, N. Impact of self-efficacy on construction labor productivity: The mediating role of work motivation. *Eng. Constr. Archit. Manag.* **32**(5), 3407–3431 (2025).

[12] Johari, S. & Jha, K. N. Impact of work motivation on construction labor productivity. *J. Manag. Eng.* **36**(5), 04020052 (2020).

[13] Wijesinghe, A. G. D. S. K., & Shantha, K. V. A. Financial incentives and employee motivation: Mediating role of workplace environment with special reference to public sector employees. (2024).

[14] Ryan, R. M. & Deci, E. L. Intrinsic and extrinsic motivations: Classic definitions and new directions. *Contemp. Educ. Psychol.* **25**(1), 54–67 (2000). 10620381 10.1006/ceps.1999.1020

[15] Bakker, A. B., Hakanen, J. J., Demerouti, E. & Xanthopoulou, D. Job resources boost work engagement, particularly when job demands are high. *J. Educ. Psychol.* **99**(2), 274 (2007).

[16] Bauer, G. F., Hämmig, O., Schaufeli, W. B. & Taris, T. W. A critical review of the job demands-resources model: Implications for improving work and health. In *Bridging Occupational Organizational and Public Health: A Transdisciplinary Approach* 43–68 (Springer, 2014).

[17] Gagné, M. & Deci, E. L. Self-determination theory and work motivation. *J. Organ. Behav.* **26**(4), 331–362 (2005).

[18] Khan, S. A. R., Zia-ul-haq, H. M., Umar, M. & Yu, Z. Digital technology and circular economy practices: An strategy to improve organizational performance. *Bus. Strateg. Dev.* **4**(4), 482–490 (2021).

[19] Etikan, I., Musa, S. A. & Alkassim, R. S. Comparison of convenience sampling and purposive sampling. *Am. J. Theor. Appl. Stat.* **5**(1), 1–4 (2016).

[20] Herzberg, F. I. *Work and the Nature of Man* (American Psychological Association, 1966).

[21] Kaynak, H. The relationship between total quality management practices and their effects on firm performance. *J. Oper. Manag.* **21**(4), 405–435 (2003).

[22] Prasanna, M. & Vinodh, S. Lean Six Sigma in SMEs: An exploration through literature review. *J. Eng. Des. Technol.* **11**(3), 224–250 (2013).

[23] Robbins, S. P., Judge, T. A. & Vohra, N. *Organizational behaviour by Pearson 18e* (Pearson Education India, 2019).

[24] Chowdhury, L. A. M., Rana, T., Akter, M. & Hoque, M. Impact of intellectual capital on financial performance: Evidence from the Bangladeshi textile sector. *J. Account. Organ. Chang.* **14**(4), 429–454 (2018).

[25] Ali, H., Khan, E. & Ilahi, I. Environmental chemistry and ecotoxicology of hazardous heavy metals: Environmental persistence, toxicity, and bioaccumulation. *J. Chem.* **2019**(1), 6730305 (2019).

[26] Rafique, A. A., Jalal, A. & Kim, K. Automated sustainable multi-object segmentation and recognition via modified sampling consensus and Kernel sliding perceptron. *Symmetry* **12**(11), 1928 (2020).

[27] Amponsah-Tawiah, K. & Mensah, J. Occupational health and safety and organizational commitment: Evidence from the Ghanaian mining industry. *Saf. Health Work* **7**(3), 225–230 (2016). 27630792 10.1016/j.shaw.2016.01.002 PMC5011093

[28] Sharma, G. D., Thomas, A. & Paul, J. Reviving tourism industry post-COVID-19: A resilience-based framework. *Tour. Manag. Perspect.* **37**, 100786 (2021). 33391988 10.1016/j.tmp.2020.100786 PMC7771910

[29] Chaudhary, R. & Panda, C. Authentic leadership and creativity: The intervening role of psychological meaningfulness, safety and work engagement. *Int. J. Product. Perform. Manag.* **67**(9), 2071–2088 (2018).

[30] Hollman, T., Palmer, N. F., Chaffin, D. & Luthans, K. Lying, cheating, & stealing: Strategies for mitigating technology-driven academic dishonesty in collegiate schools of business. *Mountain Plains J. Bus. Technol.* **22**(1), 6 (2021).

[31] Anitha, J. Determinants of employee engagement and their impact on employee performance. *Int. J. Product. Perform. Manag.* **63**(3), 308–323 (2014).

[32] Dwivedi, Y. K. et al. Impact of COVID-19 pandemic on information management research and practice: Transforming education, work and life. *Int. J. Inf. Manag.* **55**, 102211 (2020).

[33] Bakotić, D. Relationship between job satisfaction and organisational performance. *Econ. Res.-Ekonomska istraživanja* **29**(1), 118–130 (2016).

[34] Salas-Vallina, A., López-Cabrales, Á., Alegre, J. & Fernández, R. On the road to happiness at work (HAW): Transformational leadership and organizational learning capability as drivers of HAW in a healthcare context. *Pers. Rev.* **46**(2), 314–338 (2017).

[35] Manzoor, F., Wei, L., Asif, M., Haq, M. Z. U. & Rehman, H. U. The contribution of sustainable tourism to economic growth and employment in Pakistan. *Int. J. Environ. Res. Public Health* **16**(19), 3785 (2019). 31597376 10.3390/ijerph16193785 PMC6801594

[36] Tremblay, M. A., Blanchard, C. M., Taylor, S., Pelletier, L. G. & Villeneuve, M. Work extrinsic and intrinsic motivation scale: Its value for organizational psychology research. *Can. J. Behav. Sci.* **41**(4), 213 (2009).

[37] Psomas, E. & Antony, J. Total quality management elements and results in higher education institutions: The Greek case. *Qual. Assur. Educ.* **25**(2), 206–223 (2017).

[38] Sallis, E. *Total Quality Management in Education* (Routledge, 2014).

[39] Inuwa, M. Job satisfaction and employee performance: An empirical approach. *Millennium Univ. J.* **1**(1), 90–103 (2016).

[40] Lee, M. C. C., Idris, M. A. & Delfabbro, P. H. The linkages between hierarchical culture and empowering leadership and their effects on employees’ work engagement: Work meaningfulness as a mediator. *Int. J. Stress. Manag.* **24**(4), 392 (2017).

[41] Alfes, K., Shantz, A. D., Truss, C. & Soane, E. C. The link between perceived human resource management practices, engagement and employee behaviour: A moderated mediation model. *Int. J. Human Resour. Manag.* **24**(2), 330–351 (2013).

[42] Markos, S. & Sridevi, M. S. Employee engagement: The key to improving performance. *Int. J. Bus. Manag.* **5**(12), 89 (2010).

[43] Singh, A., Pal, Y., Kumar, R., Kumar, S., Bhardwaj, A., Rani, K., & Ana, R. Equine husbandry based agri-entrepreneurship-anoverview. *J. Community Mobilization Sustain. Dev*, **3**, 697–704 (2022).

[44] Ali, M., & Hassan, R. Inconsistent quality control in the leather industry: Challenges and solutions. *International Journal ofIndustrial Studies*, **12**(3), 45–58 (2018).

[45] Moos, R. H. Work Environment Scale manual (4th ed.). Consulting Psychologists Press (2008).

[46] Field, A. Discovering statistics using IBM SPSS Statistics (4th ed.). SAGE Publications (2013).

[47] Pallant, J. SPSS survival manual: A step by step guide to data analysis using IBM SPSS (6th ed.). McGraw-Hill Education (2016).

[48] Dancey, C. P., & Reidy, J. Statistics without maths for psychology (7th ed.). Pearson Education Limited (2017).

[49] Hayes, A. F. Introduction to mediation, moderation, and conditional process analysis: A regression-based approach (2nd ed.).The Guilford Press (2017).

[50] Hayes, A. F. Partial, conditional, and moderated moderated mediation: Quantification, inference, and interpretation.*Communication Monographs*, **85**(1), 4–4010.1080/03637751.2017.1352100 (2018).

[51] Smith, J. G., Morin, K. H., & Lake, E. T. Association of the nurse work environment with nurse incivility in hospitals. *Journal ofnursing management*, **26**(2), 219–226 (2018). 10.1111/jonm.12537 PMC5851800 28990326

[52] Appleton, K. M., Woodside, J. V., Yarnell, J. W. G., Arveiler, D., Haas, B., Amouyel, P., ... & PRIME Study Group. Depressedmood and dietary fish intake: direct relationship or indirect relationship as a result of diet and lifestyle?. *Journal of affective disorders*,**104**(1-3), 217–223 (2007). 10.1016/j.jad.2007.03.012 17475339

[53] Patel, P. K., Greene, M. T., Jones, K., Rolle, A. J., Ratz, D., Snyder, A., ... & Chopra, V. Quantitative results of a nationalintervention to prevent central line–associated bloodstream infection: A pre–post observational study. *Annals of internal medicine*,**171**, S23–S29 (2019). 10.7326/M18-3533 31569230

[54] Juran, J. M. Juran’s quality handbook (5th ed.). McGraw-Hill (1999).

[55] Kumari, N., Mehta, V. P., & Bhatia, J. K. (2020). Foodgrains production in India: Trend and decompositions analysis. *EconomicAffairs*, **65**(3), 333–342.

[56] Katz, H. C. The role of executive coaching in managing organizations. Human Service Organizations: Management,Leadership & Governance, **45**(2), 177–183. 10.1080/23303131.2021.1915439 (2021).

[57] Khosravi, E., Hemmatyar, A. M. A., Siavoshani, M. J., & Moshiri, B. Safe deep driving behavior detection (S3D).*Ieee Access*,**10**, 113827–113838 (2022).

